# Flexural Behavior of a Novel Textile-Reinforced Polymer Concrete

**DOI:** 10.3390/polym14010176

**Published:** 2022-01-02

**Authors:** Daniel Heras Murcia, Bekir Çomak, Eslam Soliman, Mahmoud M. Reda Taha

**Affiliations:** 1 Department of Civil, Construction & Environmental Engineering, University of New Mexico, MSC01 1070, Albuquerque, NM 87131, USA; dherasmurcia@unm.edu; 2 Department of Civil Engineering, Faculty of Engineering, Düzce University, Düzce 81600, Turkey; bekircomak@duzce.edu.tr; 3 Department of Civil Engineering, Assiut University, Assiut 71516, Egypt; esoliman@aun.edu.eg

**Keywords:** textile reinforced polymer composites, flexural mechanical characterization, ductility, crack pattern, adhesion, debonding, thermosetting polymer concrete

## Abstract

Textile reinforced concrete (TRC) has gained attention from the construction industry due to its light weight, high tensile strength, design flexibility, corrosion resistance, and remarkably long service life. Some structural applications that utilize TRC components include precast panels, structural repair, waterproofing elements, and façades. TRC is produced by incorporating textile fabrics into thin cementitious concrete panels. Premature debonding between the textile fabric and concrete due to improper cementitious matrix impregnation of the fibers was identified as a failure-governing mechanism. To overcome this performance limitation, in this study, a novel type of TRC is proposed by replacing the cement binder with a polymer resin to produce textile reinforced polymer concrete (TRPC). The new TRPC is created using a fine-graded aggregate, methyl methacrylate polymer resin, and basalt fiber textile fabric. Four different specimen configurations were manufactured by embedding 0, 1, 2, and 3 textile layers in concrete. Flexural performance was analyzed and compared with reference TRC specimens with similar compressive strength and reinforcement configurations. Furthermore, the crack pattern intensity was determined using an image processing technique to quantify the ductility of TRPC compared with conventional TRC. The new TRPC improved the moment capacity compared with TRC by 51%, 58%, 59%, and 158%, the deflection at peak load by 858%, 857%, 3264%, and 3803%, and the toughness by 1909%, 3844%, 2781%, and 4355% for 0, 1, 2, and 3 textile layers, respectively. TRPC showed significantly improved flexural capacity, superior ductility, and substantial plasticity compared with TRC.

## 1. Introduction

Textile reinforced concrete (TRC) is a cementitious composite that has been recently introduced to civil infrastructure as new precast elements and to strengthen deteriorated structures due to its lightweight, durability, design and installation flexibility, and in- and out-of-plane structural performance [1,2,3,4,5]. Typically, TRC is created by incorporating high-performance textile reinforcement made of continuous multi-filament yarns (e.g., glass, polypropylene, polyamides) into a matrix comprised of cement as a binder and small-size, well-distributed aggregate [6,7]. Planar (2D) or spatial (3D) textile reinforcement can be used in TRC depending on the load application [3,4,5,8,9]. Potential applications for TRC include architecture façade, noise/water protection panels, sandwich walls, load-bearing shell structures, storage units (e.g., tanks and silos) [1,10], and the strengthening of existing reinforced concrete structures [11,12,13,14,15,16,17,18,19,20,21]. Studies showed that TRC could replace fiber-reinforced polymer (FRP) composites for flexural and shear retrofitting with similar capacity enhancement but improved durability [22,23].

Several textile reinforcement materials have been used in TRC, including polypropylene, alkali-resistant glass, and carbon fibers. Research has investigated the flexural behavior of TRC composites. Hegger et al. [24] studied the effect of the reinforcement ratio on the flexural behavior of TRC composites using 4-point bending for carbon and glass fiber textile reinforcement materials. Silva et al. [25] investigated the bending behavior (3-point bending) of cement-based composites reinforced with continuous unidirectional sisal fibers. A multi-cracking and strain-hardening behavior was observed by the researchers for the TRC composite. Pakravan et al. [26] studied the effect of textile surface treatment on the flexural properties of TRC by carrying out 3-point bending tests for carbon fiber textile reinforced cement composites with the addition of polyvinyl alcohol (PVA) fibers. The addition of PVA fibers showed a crack bridging effect and a strain-softening behavior, thus improving the flexural capacity and energy absorption of the TRC composite. Tsesarsky et al. [27] investigated the bending behavior of TRC composites incorporating polyethylene (PE), glass, or carbon textile materials, using 3-point bending tests. TRC reinforced with carbon fibers showed the highest flexural strength and ductility. TRC incorporating PE textile exhibited a low flexural strength but high ductility. Glass fiber reinforced TRC showed intermediate strength but limited ductility.

Research investigating the use of basalt fibers as an alternative material for textile reinforcement is limited in the literature [8]. Basalt fibers can be produced from natural resources (volcanic rocks); they require low energy to manufacture, are recyclable, and have high performance while being relatively inexpensive compared with other types of fibers [28]. Due to these advantages, basalt fibers have positioned themselves as a promising material for textile and fiber reinforcement of cementitious composites [26,27]. Researchers studied the effect of the number of basalt layers on the tensile properties of TRC composites [29]. They concluded that the number of cracks increased as the number of reinforcement layers increased but the crack width and spacing would decrease. The reinforcement ratio was also concluded to have a positive effect on the crack pattern, tensile capacity and ductility for uniaxial tensile testing conditions [30]. Du et al. [8] investigated the influence of the number of reinforcing layers, the level of prestress of the textile and the influence of the presence of steel fibers on the flexural behavior of basalt reinforced TRC using 4-point bending tests. The increase in the number of textile layers was shown to improve the flexural capacity and the toughness. Similar results were reported by Zhu et al. [31] for glass fiber reinforcement. It was concluded that the improvement in the energy absorption capacity of the TRC composite is strongly dependent on the number of reinforcement layers.

In this context, TRC might be sought as an alternative to concrete reinforced with discrete short, chopped fibers known as fiber reinforced concrete (FRC). In FRC, short fibers (typically steel) have been used to reinforce plain concrete. Discrete short fibers mainly provide crack control due to their ability to transfer tensile stresses across crack surfaces known as crack-bridging [32]. Therefore, FRC members demonstrate a pseudo-ductile response, increased residual strength, especially in tension and flexure, and enhanced energy dissipation capacities relative to the brittle behavior of plain concrete. Furthermore, fibers have been proved as a promising non-conventional mass reinforcement under shear stresses and, and under specific circumstances, could alter the brittle shear failures to ductile flexural ones [32,33,34]. Development of TRC will be guided by the collective knowledge and research experiences accumulated on FRC over the past decades.

Despite its unique structural performance, research has unveiled a few challenges related to the failure behavior of TRC structural elements—specifically, the potential for premature debonding between the textile yarns and the cement matrix at low strains. Organic coats were suggested to improve the bond behavior between the fibers and the mortar [35]. Previous research showed that such early debonding could be attributed to the improper impregnation of the fiber fabrics by the cementitious matrix. Microscopic images showed that the degree of fiber impregnation varies within fiber filaments, with outer (sleeve) filaments being fully impregnated and fiber impregnation decrease toward the central (core) fibers [7,36]. The high viscosity of the cement slurry contributes to the low penetration of the cement matrix into narrow spaces in the textile yarn. However, stresses can still be transferred to the core fiber layers via frictional contact with the adjacent outer fiber layers [37,38]. When TRC is subjected to high loading rates (e.g., impact or dynamic), further debonding between the reinforcement textile layers and the cement matrix occurs. Debonding may lower the load-carrying capacity and ductility, potentially altering the failure mode of the TRC structure [38].

On the other hand, polymer concrete (PC) is a composite material developed by combining a polymer resin that fully substitutes the cement as a binder and well-graded aggregate particles as a filler [39]. Polymers such as epoxy, polyester, vinyl ester, and methyl methacrylate (MMA) are commonly used in the construction industry [40]. The PC mixture typically contains an initiator to promote polymerization and to control the hardening time. Combining a polymer matrix and aggregate enables researchers and contractors to engineer specific desired properties due to the composite’s physicochemical properties such as low permeability, high ductility, high durability, chemical resistance, and good mechanical strength [24,25]. Aggregate type is chosen to achieve desired characteristics such as high strength [41,42,43,44], thermal stability [45,46,47,48], fire resistance [49,50,51], electrical conductivity [46,47,52], or electromagnetic shielding [53]. Polymers are also known for their significantly higher bond strength to many surfaces compared with cement. PC is, therefore, favorable when a strong bond to the reinforcement is required. Research [54,55,56] has shown how the mass reinforcement of epoxy polymer concrete with discrete short carbon or glass fibers increased the flexural capacity and fracture toughness compared with unreinforced specimens. The mechanism behind this improvement was found to be the good bonding between the matrix and the fibers [56]. Furthermore, research has studied how the combination of high stiffness (steel) and low stiffness (macro-synthetic) fibers affects the fresh properties of fiber reinforced concrete (FRC) composites [57]. Researchers showed that particulate-filled epoxy polymer has high durability and mechanical characteristics suited to use as a protective coating and in building applications [58]. PC reinforced with discrete short fibers and textile reinforced polymer concrete (TRPC) as composite materials have the potential to overcome some of the limitations of polymer concrete and can be used for applications that require high tensile strength, high cracking resistance, high fatigue resistance, high ductility and freeze-thaw resistance. Applications that demand these requirements include but are not limited to composite railway sleepers [56], bridge deck overlays, bridge deck closure joints, impact resistant structures, underground utility boxes, and thin PC elements made using additive manufacturing [40,59].

Researchers studied the mechanical properties of textile reinforced cement-based composites and identified limitations inherent to the cement-textile bond mechanism. To overcome the above-mentioned limitations, this study examines the flexural behavior of an innovative textile reinforced polymer concrete (TRPC) that is produced using basalt fiber textile fabrics, well-graded fine aggregate, and MMA polymer as a binder. This is the first study to examine a polymer-based textile reinforced concrete composite as an alternative to conventional cement-based textile composites. Flexural testing was conducted on TRPC with different reinforcement configurations. The flexural capacity, ductility, toughness, and crack pattern of TRPC were quantified and compared with conventional TRC, fabricated using Portland cement concrete of similar strength and with the same textile fabric and reinforcement configurations.

## 2. Materials and Methods

### 2.1. Materials

**Figure 1:**
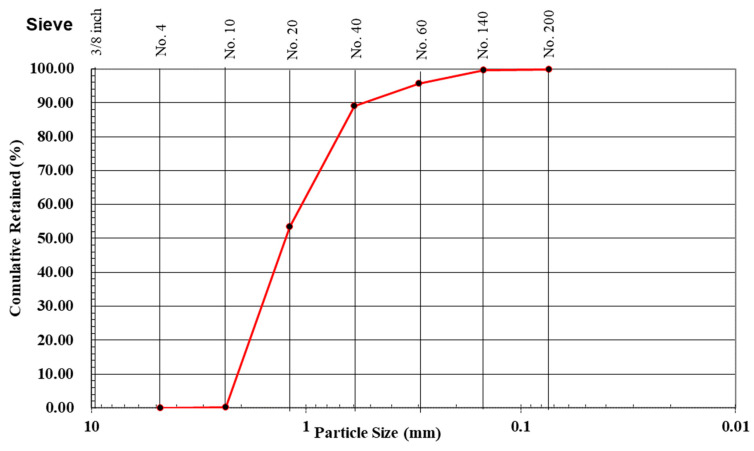
Sieve size distribution for aggregate used in both TRC and TRPC mixes.

MMA polymer (Transpo Industries, New Rochelle, NY, USA) was used to produce the PC. The polymer system consists of two parts: a low viscosity MMA monomer resin and Benzoyl Peroxide as an initiator. Type I/II Portland cement and a water/cement ratio of 0.25 were used for the conventional concrete mix. BASF Master Glenium 3030 (BASF, Florham Park, NJ, USA) superplasticizer was used with the conventional concrete mix. A well-graded fine quartz aggregate with a nominal maximum size of 2.36 mm was used for both cement concrete and PC mixes. The aggregate size distribution is depicted in Figure 1.

The nominal maximum size of the aggregate needed to be smaller than the 5 mm window size of the textile fiber fabric to ensure proper impregnation. The concrete mix proportions for both the PC mix (used for TRPC) and the reference conventional concrete mix (used for TRC) are presented in Table 1. As per ASTM C1437-20 [60], the flow values for both TRC and TRPC mixes are 113% and greater than 160% respectively. The cement concrete reference specimens were cast and cured in a standard curing room at 99% relative humidity (RH) and 22 °C for 28 days to reach the design strength. Studies reported that PC achieves about 75% of its strength within one day of curing [61,62,63] and most of its full strength after seven days of curing in such ambient temperatures [64]. PC was therefore left to cure in the lab ambient conditions of 30% RH and 22 °C for seven days [40]. Five cylinders, 2 in (50.8 mm) diameter by 4 in (101.6 mm) height, of each mix were cast to determine the compressive strength of conventional concrete and PC as per ASTM C39-17b [65]. The mean compressive strength for the conventional reference concrete after 28 days was 68.9 ± 5.1 MPa, and the average compressive strength of PC mixture was 53.9 ± 5.8 MPa. The cement-based concrete mix was intentionally designed to have similar or higher compressive strength than the polymer-based mix to ensure equitable comparison. A bidirectional planar basalt fiber textile mesh (Smarter Building Systems, New Port, RI, USA) with no surface treatment was used for the reinforcement of both TRC and TRPC specimens. The characteristics of the basalt fiber textile mesh, as reported by the manufacturer, are presented in Table 2.

### 2.2. Mixing and Casting

**Figure 2:**
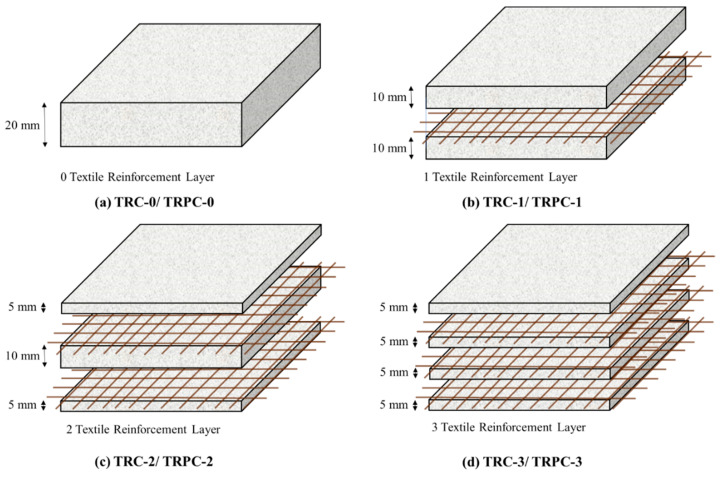
Schematic representation of the fabricated TRC and TRPC panels incorporating zero (**a**), one (**b**), two (**c**), and three (**d**) basalt fiber textile reinforcement layers.

Reference conventional concrete and PC mixtures were mixed using the proportions described above. Both mixes were mixed for 3–5 min using a 4.7-L bowl-lift standard mixer until a uniform mixture was obtained. Then, TRC and TRPC panels of 400 × 144 × 20 mm (length × width × thickness) were cast using the conventional concrete or PC mixture, respectively, and the basalt fiber textile fabrics described above. Four configurations of TRPC panels were fabricated by incorporating 0, 1, 2, and 3 layers of basalt fabric denoted as TRPC-0, TRPC-1, TRPC-2, and TRPC-3 respectively. The same reinforcement configurations were used to fabricate the reference TRC panels, denoted as TRC-0, TRC-1, TRC-3, and TRC-3. The placement of the reinforcement mesh at the neutral axis location for specimens TRC-1 and TRPC-1 was selected so that the effect of the reinforcement in the post-peak response could be observed. Figure 2 illustrates the configurations of TRPC and TRC panels.

**Figure 3:**
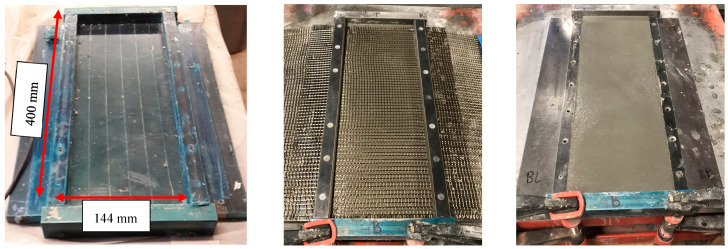
Process for the fabrication of TRC and TRPC panels. (**Left**) Mold used for fabrication. (**Middle**) Addition of an intermediate basalt fiber textile fabric above the concrete or PC layer. (**Right**) TRC panel after fabrication.

All TRC/TRPC panels, regardless of the number of textile reinforcement layers used, were cast in four layers following a layer-by-layer method. A layer of fresh concrete (or PC) was placed first, and the basalt fiber textile fabric was placed at the desired thickness location afterward. The second layer of fresh concrete (cement or polymer-based) was cast, and the process was repeated until the total thickness of the specimen was obtained. Figure 3 shows the fabrication setup of the TRPC panels. All TRC and TRPC panels were demolded after 24 h and then cured using the two curing protocols described above.

### 2.3. Flexure Test Setup

**Figure 4:**
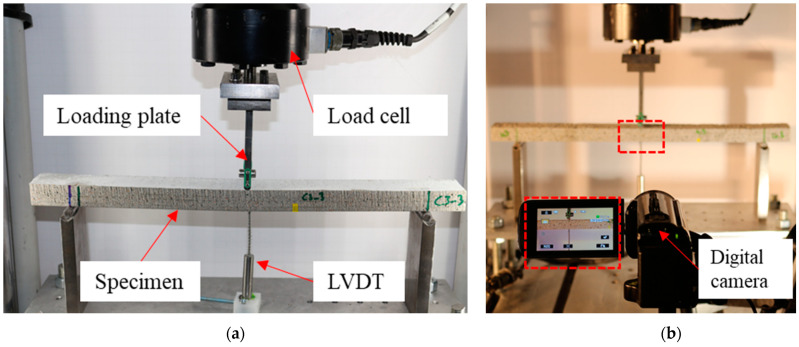
Three-point bending test setup. (**a**) Specimen during testing and LVDT displacement recording system; (**b**) Digital image data acquisition system.

The TRC/TRPC panels were cut into 400 mm × 35 mm × 20 mm (length × width × thickness) specimens using a waterjet cutting machine. The prisms were tested in three-point bending with a span length of 330 mm, achieving a span-to-depth ratio of 16.5. TRC composites are typically used for reinforcement of existing structures or for precast elements for façade or shell structures. Most of these structures are typically very thin elements [24]. The 20 mm specimen thickness was chosen to simulate thin TRC element. The selection of the span-to-depth ratio is congruent with the literature [8,66,67,68]. The span-to-depth ratio selected is also suitable given the relatively high deformations reported for TRC specimens and since most of the TRC applications are for shell-like structures [1,2,6,7,24]. The deflection at the midspan location was measured using a linear variable differential transducer (LVDT). The load and displacement of the load cell were also recorded using MTS^®^ Bionix servo-hydraulic system with a range of 0–25 kN and a resolution of 1 N. For testing, a displacement rate of 0.5 mm/min was imposed at the mid-span location. Three specimens were tested for each TRC/TRPC reinforcement configuration. The test setup is shown in Figure 4a. The three-point bending test setup included the digital image acquisition system to capture crack patterns during testing. The crack pattern intensity was quantified via digital image processing technique to assess the ductility of TRC/TRPC. Photoshop^®^ and ImageJ^®^ software were employed for examining the cracking pattern. Details for the method used for image transformation and processing have been described elsewhere [69]. Two types of cracks were analyzed, horizontal and vertical cracks, to assess the flexural ductility and the concrete-fabric bond strength. Figure 4b shows the digital image data acquisition.

## 3. Results and Discussion

**Figure 5:**
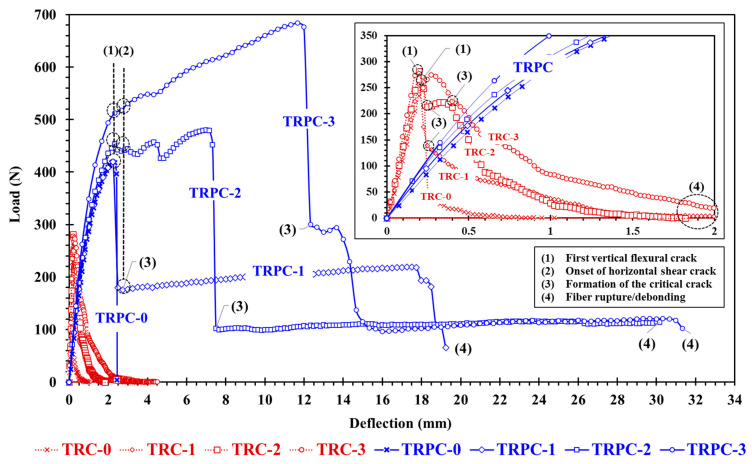
Load-displacement response of TRPC specimens with the different number of textile layers compared with TRC. Three specimens were tested for each case. One median test specimen is shown for each type for clarity. Inset shows a close-up to show the behavior of TRC.

**Figure 6:**
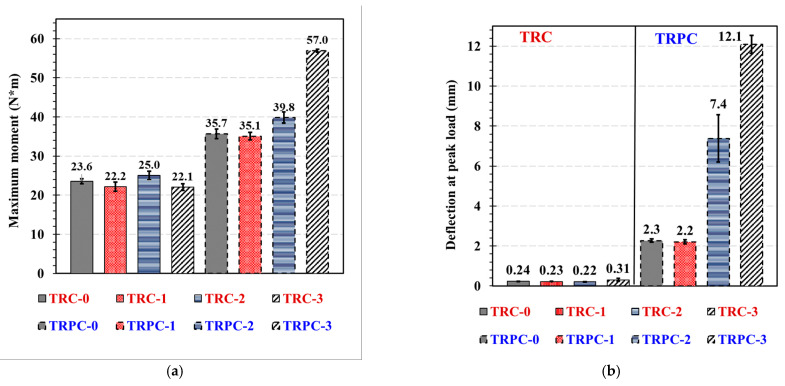

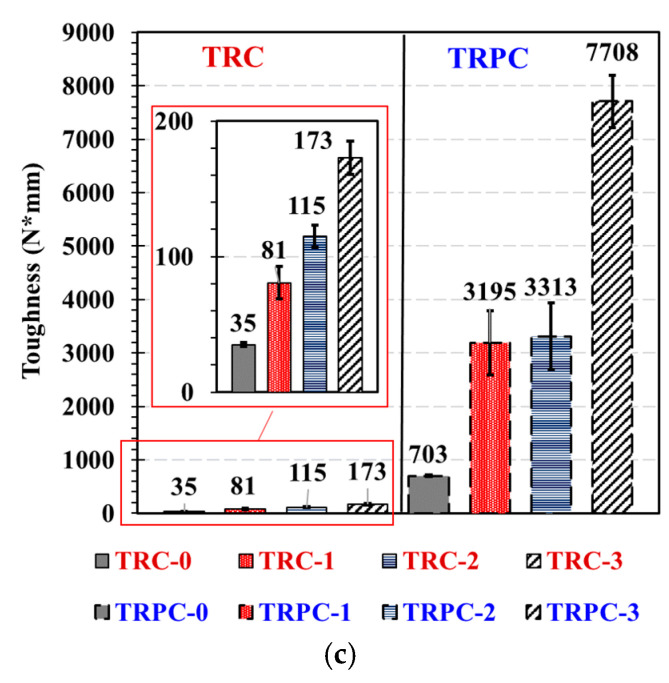
(**a**) moment, (**b**) deflection at maximum load, and (**c**) toughness, measured up to a post-peak residual capacity of 25% of the maximum load for TRC and TRPC with different reinforcement layers.

The median load-deflection curves of the three-point bending tests of TRC and TRPC specimens with different textile layers are shown in Figure 5. The mean flexural strength, deflection at maximum load, and toughness (energy absorption up to a post-peak residual capacity of 25% of the maximum load) of TRC and TRPC for the different reinforcement configurations are presented in Figure 6. To examine the statistical significance while comparing the means of two sets of data, the Student’s T-test (*t*-test) was applied. A 95% confidence interval was used (α = 0.05) for all the T-tests. Du et al. [8] reported similar mechanical properties in terms of first-crack flexural stress of 7 MPa for TRC specimens compared to 7.9 MPa for TRC-3 specimens reported herein. The stress is calculated from the peak-load assuming linear-elastic behavior up to the first crack load (TRPC exhibits a non-linear behavior after this load).

Figure 5 and Figure 6 show the superior mechanical performance of TRPC in terms of flexural strength, deflection at peak load, and toughness, for all the TRPC specimens compared with conventional TRC. The improvement in the mechanical behavior can be attributed to the higher tensile capacity and ductility of the polymer (as depicted by comparing TRC-0 to TRPC-0) and its better bond with the basalt fibers compared with cement paste. Descriptors (1–4) in Figure 5 identify: (1) first vertical flexural crack, (2) onset of horizontal shear crack, (3) formation of the critical crack, (4) fiber rupture/debonding. The figures also demonstrate the fact that the behavior of reinforced and unreinforced TRPC specimens is significantly different compared with TRC. For TRC specimens, the addition of textile reinforcement layers did not significantly (*t*-test, α = 0.05) affect the maximum moment capacity or deflection at peak load (23.6, 22.2, 25, 22.1 N.m for TRC-0, TRC-1, TRC-2, and TRC-3, respectively). Similar behavior in terms of first-crack flexural stress was observed by Du et al. [8] for TRC with 3 and 4 layer configurations. This can be explained by the limited bond strength between the fibers and the cementitious matrix due to the high viscosity of the cement slurry and, consequently, the low penetrability of the cement matrix to the fiber mesh. Failure due to premature debonding between the textile yarns and the cement matrix at low strains has been widely reported in the literature [7,35,36,37,38]. On the other hand, the difference in terms of toughness for the TRC configurations is statistically significant (*t*-test, α = 0.05) due to the difference in post-peak behavior between the reinforcement configurations.

The addition of two and three textile layers in the TRPC specimens significantly increased the maximum flexural strength (especially for TRPC-2 and TRPC-3) compared with TRPC-0. The use of two and three layers of basalt textile fabric improved the maximum moment capacity of TRPC-2 and TRPC-3 by 11.5% and 59.5%, respectively, compared with TRPC-0. Similar behavior is observed for the deflection at peak load, which increased by 225% and 432% for TRPC-2, and TRPC-3, respectively, compared with TRPC-0. The moment capacity of the TRPC-3 specimen was superior when compared to TRPC-2 even though the third textile layer was placed at the neutral axis. This is because both specimens start behaving nonlinearly from the 400 N load due to the material nonlinear behavior of the polymer concrete mix. Nonlinear behavior of polymer concrete beams has been widely reported in the literature [70,71,72]. This material non-linearity causes a shift in the neutral axis position and will cause the middle textile layer of TRPC to bear load. This behavior is not observed for the TRC-2 and TRC-3 specimens due to the linear elastic nature of the cement-based concrete material. Nonetheless, Du et al. [8] was able to observe a nonlinear behavior for TRC composites using a four point-bending configuration (rather than the 3-point bending one used herein). Statistical analysis shows that the difference in performance for TRPC-1 compared to TRPC-0, in terms of the maximum moment, is not statistically significant (*t*-test, α = 0.05). This is due to the low efficiency of the reinforcement when placed at the neutral axis position. For the textile reinforcement to be loaded, the neutral axis would need to move towards the top-half of the composite cross-section. For this to happen, the specimen needs to crack, and thus no reinforcement contribution is provided prior to the first-crack state. Other researchers reported on this phenomenon [8,68]. Nevertheless, the post-peak behavior, after cracking, is significantly improved as the textile reinforcement carries the tension stresses and allows the TRC composite to continue carrying load as shown in Figure 5. The significance of the textile reinforcement is reflected in the improved toughness depicted in Figure 6c.

To assess the effect of fabric layers on the post-peak mechanical behavior of the panels, the flexural toughness was quantified and compared. The results show significant improvement of TRPC energy absorption by 354%, 371%, and 996% with the addition of one, two, and three basalt-fiber layers. The significant improvement in ductility/energy absorption depends on the improved load and deflection capacity and on the TRPC panels’ ability to continue carrying the load after exceeding the maximum capacity. The values obtained for TRPC-3 (7.7 N·m) are comparable to the ones obtained by other researchers for cement-based TRC reinforced with chopped steel fibers and textile prestressing (6.7 N·m for the optimal steel fiber content and prestress level) [8]. Figure 5 shows that as the number of fabric layers increases, the residual load capacity of TRC/TRPC increases. For TRC, the toughness also improves as the number of basalt-textile layers is increased. The use of 1, 2, and 3 layers of basalt fiber textile fabric resulted in improving the toughness of TRC-1, TRC-2, and TRC-3 by 131%, 228%, and 394%, respectively, compared with TRC-0. It is apparent that the improvement in toughness for TRC is much more limited compared with TRPC. It is also evident that the mechanical behavior of the novel TRPC outperforms TRC for all the reinforcement configurations.

**Figure 7:**
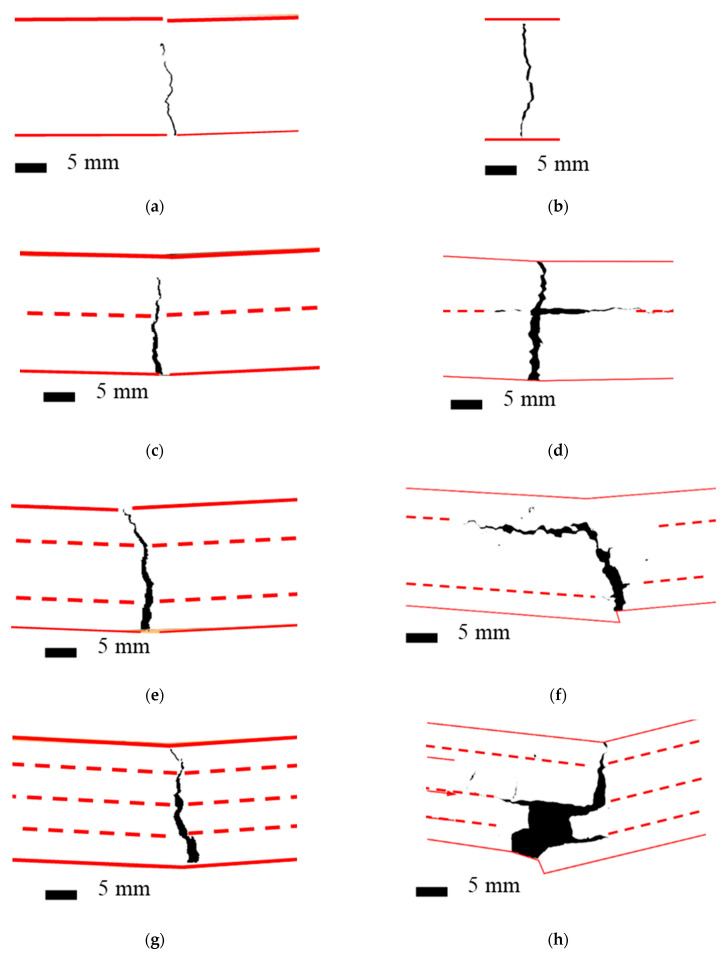
Cracking pattern for TRC and TRPC specimens with different reinforcement configurations recorded using DIC on the side of the specimens. Red continuous lines represent the contours of the specimens. Dotted marked lines represent the location of the textile reinforcement. (**a**) TRC-0 (**b**) TRPC-0 (**c**) TRC-1 (**d**) TRPC-1 (**e**) TRC-2 (**f**) TRPC-2 (**g**) TRC-3 (**h**) TRPC-3.

The crack pattern in TRC and TRPC specimens was analyzed using a digital image correlation system (DIC) for both horizontal and vertical cracks to assess cracking intensity and ductility. DIC has been used by many authors to measure crack opening and growth for concrete materials [73,74,75]. Crack pattern analysis results for horizontal and vertical cracks are presented in Table 3 and Table 4. Moreover, examples of crack patterns for different TRC and TRPC specimens are shown in Figure 7.

**Figure 8:**
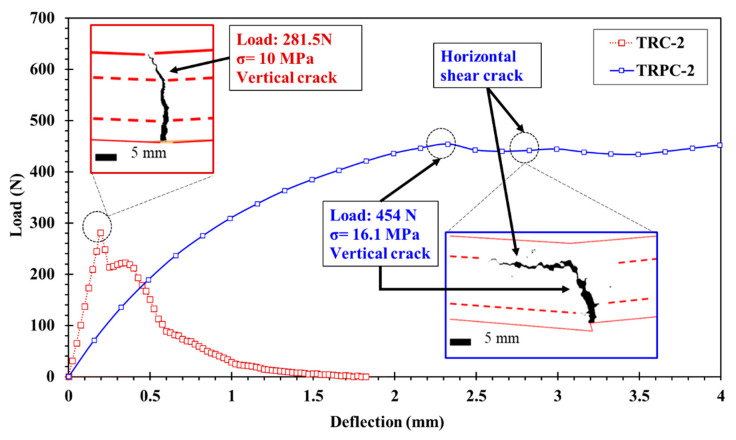
Load-displacement response of TRPC-2 specimen compared with TRC-2 specimen. Three specimens were tested for each case. One median test specimen is shown for each type for clarity. Inset pictures show a close-up view of the cracking behavior of the composite and the first-crack load.

Table 3 and Figure 7 show that no horizontal cracks were observed for the TRC reinforcement configurations and TRPC-0 configurations. This indicates that the vertical cracks grow relatively unrestricted with the absence of intermediate reinforcement. This is also an indication of the relatively low plasticity of TRC and TRPC without any textile reinforcement due to the absence of a mechanism to divert and deviate crack propagation. On the other hand, horizontal cracks were observed in all TRPC specimens incorporating textile reinforcement. The propagation of horizontal cracks delayed failure and thus provided plastic and ductile behavior. Horizontal cracks were only observed in TRPC and not in TRC. This can be attributed to the fact that TRPC can resist higher tension stresses than TRC and therefore was able to develop high shear stresses at the fiber yarn-matrix interface. This high shear stress would ultimately result in the development of horizontal shear cracks. These horizontal cracks were not observed in TRC which fails prematurely under tensile stresses due to the relatively low-tension capacity of the cement matrix. This phenomenon is depicted in Figure 8 for the two-layer configuration textile reinforced composites (TRC-2 and TRPC-2).

The development of horizontal cracks also demonstrates the ability of the textile fabric to improve fracture toughness for TRPC specimens compared with conventional TRC. The resistance to crack extension could be attributed to the good bond strength between the basalt textile fabric and PC. In TRC, the limited bond strength between the basalt textile fabric and cement matrix leads to lower cracking strength, resistance to crack propagation, and flexural capacity compared with TRPC. It is also apparent that the width of horizontal cracks depends on the number of textile reinforcement layers for the TRPC specimens. As the number of textile layers increased, the average crack width increased, demonstrating the ability of TRPC to sustain residual load while having a considerable level of damage in the specimen. Similar behavior was observed for discrete fiber reinforced cement composites [76]. For instance, the average crack width of TRPC-2 was 0.45 mm, which is about twice the average crack width of TRPC-1 of 0.24 mm, and the average crack width of TRPC-3 was 0.75 mm, which is about three times that of TRPC-1.

The assessment for vertical cracks is presented in Table 4. It can be observed that the average vertical crack width increases as the number of fabric layers increase for both TRC and TRPC systems. The crack area also increased by increasing the number of textile layers. It is worth mentioning that TRPC-3 observed an average width of vertical crack of 2.4 mm, 8 times the average crack width of 0.29 mm observed for TRPC-0. In addition, the crack area of 113.8 mm^2^ for TRPC-3 was about 24 times of 4.8 mm^2^ observed for TRPC-0. The use of three layers of basalt fiber provided superior strength and ductility to TRPC.

**Figure 9:**
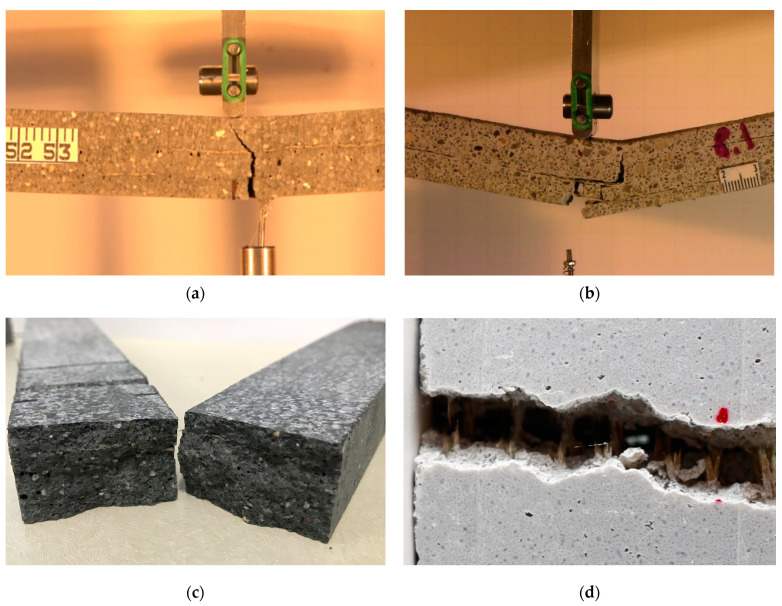
(**a**) Brittle failure of TRC-3. (**b**) Ductile failure of TRPC-3. (**c**) Close-up image of the side view of TRC-3 specimen after failure showing fiber yarn failure. (**d**) Close-up image of a bottom view of the TRPC-3 showing rupture of fiber yarns leading to the formation of wide cracks in TRPC.

It is remarkable to compare the performance of TRPC cracking in terms of vertical crack width and area to that of TRC. The vertical crack width for TRPC-1, TRPC-2, and TRPC-3 is 79%, 183%, and 182% higher than the vertical crack width observed for TRC-1, TRC-2, and TRC-3, respectively. The vertical crack area for TRPC-1, TRPC-2, and TRPC-3 is 185%, 218%, and 538%, respectively, higher than the vertical crack area observed for TRC-1, TRC-2, and TRC-3. It is important to note that the cement-based mix has a compressive strength 28% higher than the polymer-based mix. However, TRPC shows a much-improved flexural behavior regarding vertical cracking for all reinforcement configurations compared with TRC. This is due to the high tensile strength and ductility of TRPC as well as to the improved mechanical behavior at the matrix-yarn interface. To further understand the failure mechanisms of TRPC and TRC, images of failed specimens were analyzed, as shown in Figure 9.

**Figure 10:**
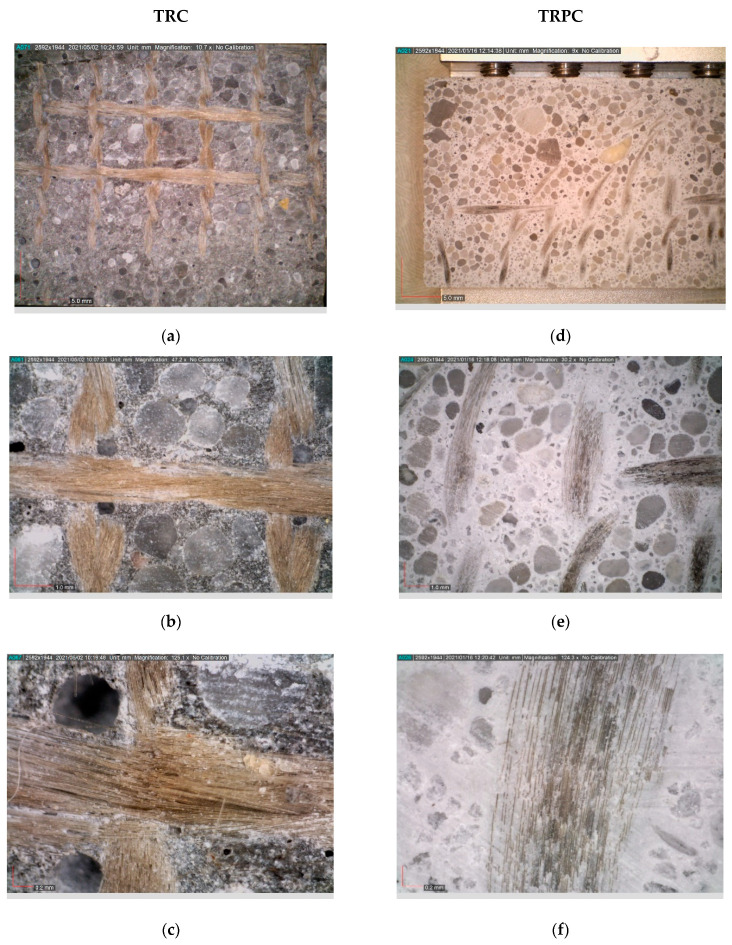
Light-microscopy pictures at a sagittal cut (through the textile-matrix interface) at three different resolutions (macro to micro-scale with left bottom corner image bar showing 5.00 mm, 1.0 mm, and 0.2 mm) for TRC (**a**–**c**) and TRPC (**d**–**f**).

The difference in failure mechanisms between TRC and TRPC is shown in Figure 9a,b. The images show that a vertical crack propagated the whole depth of textile reinforced TRC. However, the textile reinforcement in TRPC diverted the crack and resulted in a horizontal crack that allowed the specimen to continue carrying loads while demonstrating a plastic behavior. The progressive crack propagation in the case of TRPC is also evident from the load-displacement curves shown in Figure 5. Figure 9c,d shows close-up images of the failure location of TRC-3 and TRPC-3, respectively. The fiber bridging mechanism in TRPC has contributed to improving the load-carrying capacity, deformation capacity, ductility, and plasticity of the TRPC panels. This phenomenon has been reported for discrete steel fiber reinforcement cement composites [76] and modelled numerically by using a probabilistic analysis model [77]. It is hypothesized that the strong fiber bridging and yarn failure in TRPC stems from a good bond strength between the textile fabric and the polymer matrix compared with the weak bond between the textile fabric and the cement matrix as reported by others in TRC [38]. To investigate this behavior, sagittal cuts using a precision saw with a wafering diamond blade were made in the non-failed sections of the beam specimens. The specimens at the interface between the textile and matrix were analyzed using light microscopy. The light microscopic pictures of the sections for TRC and TRPC specimens for three different resolutions are depicted in Figure 10.

**Figure 11:**
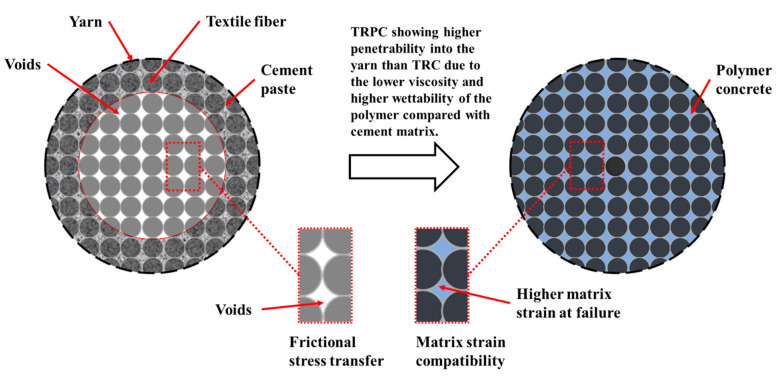
Leading factors affecting the mechanical behavior at the matrix-yarn interface for TRC/TRPC composites.

It can be observed from the above images how for the TRPC composite, the polymer matrix impregnates the fiber yarns very well (Figure 10f) compared with TRC (Figure 10c), where an apparent absence of impregnation can be observed. The ability of the fresh slurry (matrix) to impregnate the textile fabric is a critical factor that affects the bond strength between the polymer or cement matrix and the textile fabric. It is evident that the polymer material has a much higher flowability (>160% as per ASTM C1437-20 [37]) than the cement paste (113% as per ASTM C1437-20 [37]). This is due to a much lower viscosity of the polymer material (8 cps) while compared with the cement slurry (40 cps) [78] resulting in much-improved impregnation of the polymer to the textile fabric compared with the cement paste This concept is schematically presented in Figure 11. Previous research showed that improper impregnation of textile fabric associated with the use of cementitious matrix in conventional TRC caused properly impregnated outer fiber filaments and poorly impregnated central filaments [7,36]. This limitation is overcome using the novel TRPC. The improper impregnation of fiber filaments leads to reduced contact surface area between the matrix and the fibers, limiting the bond strength and resulting in limiting the fibers’ ability to bridge cracks. Fiber debonding rather than fiber rupture becomes the primary failure mechanism in TRC. In contrast with TRPC, the polymer’s low viscosity and high wettability compared with cement slurry [78] result in textile fabric impregnation along the full section of the basalt yarn. Good impregnation of the textile fibers improves the bond strength and leads to improved fiber crack bridging and enhanced flexural strength and resistance to crack propagation. Basalt yarn rupture is shown in Figure 7d as proof of the relatively high bond of basalt textile and the polymer matrix. The improved impregnation and bond also allow the TRPC to make use of the polymer matrix’s high failure strain, thus resulting in significant improvement in TRPC ductility compared with TRC. The above difference in impregnation and bond between the matrix and the textile fabric results in a difference in the stress transfer in TRPC compared with TRC. In TRC, shear forces are typically transferred between the fibers and cementitious matrix via friction due to poor adhesion. On the other hand, TRPC relies on the superior adhesion between fibers and the polymer matrix to transfer the shear forces and stress transfer through friction.

The above experimental investigations showed the improved flexural performance of TRPC represented by improved strength and superior ductility and plasticity compared with TRC. Further research is warranted to identify the optimal location and number of the textile fabrics in the composite. Furthermore, the significance of the fiber types or density was not examined. Other types of less expensive fibers than basalt fibers (e.g., bamboo fibers) can also be investigated. Finally, the ability to combine basalt fibers and sustainable polymers to produce a new sustainable TRPC shall be considered in future research.

## 4. Conclusions

The flexural behavior of innovative TRPC composite panels was examined. To manufacture the specimens, PC incorporating a different number of basalt fiber textile fabric layers was used. The flexural behavior of the new TRPC was compared with a reference TRC fabricated using conventional cement concrete with similar strength and identical reinforcement configurations, using the same basalt fiber textile fabric. Flexural load-displacement curves, maximum moment capacity, deflection at peak load, and toughness were obtained and compared. In addition, crack patterns of damaged specimens were examined via image processing techniques. TRPC panels incorporating basalt textile reinforcement exhibited a higher moment capacity of 51%, 58%, 59%, and 158%, remarkable plasticity represented by deflection at peak load increase of 858%, 857%, 3264%, and 3803%, and a substantially enhanced toughness increase of 1909%, 3844%, 2781%, and 4355% for 0, 1, 2, and 3 textile layers, respectively, when compared with conventional cementitious TRC. The flexure performance of TRPC improves as the number of reinforcement layers increases.

Cracking pattern analysis showed the ability of reinforced TRPC to develop significantly wider horizontal and vertical cracks and maintain structural integrity at high loads and deformation compared with conventional cementitious TRC. The fiber bridging effect was evident in TRPC with two and three reinforcement layers. On the other hand, limited improvement in flexure performance is achieved in TRC by incorporating basalt-textile reinforcement. The main difference in behavior between TRPC and TRC is attributed to the high bond strength between the polymer matrix and the textile reinforcement yarns compared with that of the cement matrix. The improved bond of the polymer matrix is attributed to low viscosity and high flowability, and penetrability of the polymer into the fibers of the yarns, compared with the cement paste. Such high penetrability enables TRC to experience fiber rupture rather than the typical fiber debonding observed in TRC.

Finally, it is concluded that polymer materials represent a novel and promising alternative to traditional cement-based materials for textile-reinforced concrete composites. TRPC overcomes some key limitations of TRC, positioning itself as a viable alternative material for applications that require high flexural strength and flexural toughness. This study shows that TRPC panels using basalt fiber exhibit significantly improved flexural capacity, good plasticity, and high ductility when compared with traditional cementitious TRC panels. The presented research shows that TRPC provides a significantly improved flexural behavior for all textile reinforcement configurations. The presented TRPC provides a novel composite material for the current state of practice. TRPC will be used by industry for relatively thin structural elements with high flexural strength and ductility demands, such as blast resistance panels and shell elements serving in aggressive environments (e.g., offshore structures). The proposed TRPC can also provide an excellent alternative for structural retrofitting of concrete structures in challenging service conditions (e.g., concrete manholes).

## Figures and Tables

**Table 1:** Concrete mixture proportion (kg/m^3^) for the reference conventional concrete and PC.

	Reference Conventional Concrete	PC
Portland Cement	689	-
Water	172	-
Superplasticizer	21	-
Aggregate	1378	2002
Polymer Resin	-	251

**Table 2:** Geometry, density, and mechanical characteristics of basalt fiber textile mesh.

Mesh Window Size	5.00 mm	Maximum Load-Weft	45,000 N/m
Total Weight/Area	220 g/m^2^	Elongation at break-Warp	6.7%
Weight Resin Coating	No resin coating	Elongation at break-Weft	3.5%
Thickness	0.6–0.7 mm	Breaking Elongation-Warp	13.5 mm
Maximum Load-Warp	48,000 N/m	Breaking Elongation-Weft	7.1 mm

**Table 3:** Horizontal crack length and width (mm) for TRC and TRPC (mean values).

Specimen	Horizontal Crack Length * (mm)	Horizontal Crack Width * (mm)
TRC-0	No horizontal crack	
TRC-1		
TRC-2		
TRC-3		
TRPC-0		
TRPC-1	34.6	0.24
TRPC-2	51.3	0.45
TRPC-3	27.5	0.75

* Coefficient of variation (COV < 31%).

**Table 4:** Vertical crack area (mm^2^) and width (mm) for TRC and TRPC (mean values).

Specimen	Vertical Crack Area * (mm^2^)	Vertical Crack Width * (mm)
TRC-0	4.28	0.39
TRC-1	9.46	0.58
TRC-2	13.42	0.65
TRC-3	17.81	0.85
TRPC-0	4.8	0.29
TRPC-1	27.0	1.04
TRPC-2	42.7	1.84
TRPC-3	113.8	2.40

* Coefficient of variation (COV < 34%).

## References

[1] Kulas C. (2015). Actual Applications and Potential of Textile-Reinforced Concrete. GRC.

[2] Lepenies I.G., Richter M., Zastrau B.W. (2008). A Multi-Scale Analysis of Textile Reinforced Concrete Structures. PAMM.

[3] Volkova A., Paykov A., Semenov S., Stolyarov O., Melnikov B. (2016). Flexural Behavior of Textile-Reinforced Concrete. MATEC Web Conf..

[4] Žák J., Štemberk P., Vodička J. (2017). Production of a Textile Reinforced Concrete Protective Layers with Non-Woven Polypropylene Fabric. IOP Conf. Ser. Mater. Sci. Eng..

[5] Gries T., Raina M., Quadflieg T., Stolyarov O. (2016). Manufacturing of Textiles for Civil Engineering Applications. Textile Fibre Composites in Civil Engineering.

[6] Triantafillou T. (2016). Textile Fibre Composites in Civil Engineering.

[7] Peled A., Bentur A., Mobasher B. (2017). Textile Reinforced Concrete.

[8] Du Y., Zhang X., Zhou F., Zhu D., Zhang M., Pan W. (2018). Flexural Behavior of Basalt Textile-Reinforced Concrete. Constr. Build. Mater..

[9] Sharei E., Scholzen A., Hegger J., Chudoba R. (2017). Structural Behavior of a Lightweight, Textile-Reinforced Concrete Barrel Vault Shell. Compos. Struct..

[10] Abdel-Emam M., Soliman E., Nassr A., Khair-Eldeen W., Abd-Elshafy A., Taha M.M.R. (2018). Dynamic Behavior of Textile Reinforced Polymer Concrete Using Split Hopkinson Pressure Bar. Proceedings of the International Congress on Polymers in Concrete (ICPIC 2018).

[11] Williams Portal N., Lundgren K., Wallbaum H., Malaga K. (2015). Sustainable Potential of Textile-Reinforced Concrete. J. Mater. Civ. Eng..

[12] Brückner A., Ortlepp R., Curbach M. (2006). Textile Reinforced Concrete for Strengthening in Bending and Shear. Mater. Struct..

[13] Schladitz F., Frenzel M., Ehlig D., Curbach M. (2012). Bending Load Capacity of Reinforced Concrete Slabs Strengthened with Textile Reinforced Concrete. Eng. Struct..

[14] Tetta Z.C., Koutas L.N., Bournas D.A. (2015). Textile-Reinforced Mortar (TRM) versus Fiber-Reinforced Polymers (FRP) in Shear Strengthening of Concrete Beams. Compos. Part B Eng..

[15] Papanicolaou C.G., Triantafillou T.C., Papathanasiou M., Karlos K. (2008). Textile Reinforced Mortar (TRM) versus FRP as Strengthening Material of URM Walls: Out-of-Plane Cyclic Loading. Mater. Struct..

[16] De Santis S., de Felice G. (2015). Tensile Behaviour of Mortar-Based Composites for Externally Bonded Reinforcement Systems. Compos. Part B Eng..

[17] Wang J., Wan C., Zeng Q., Shen L., Malik M.A., Yan D. (2020). Effect of Eccentricity on Retrofitting Efficiency of Basalt Textile Reinforced Concrete on Partially Damaged Masonry Columns. Compos. Struct..

[18] Truong B.T., Bui T.T., Limam A., Si Larbi A., Le Nguyen K., Michel M. (2017). Experimental Investigations of Reinforced Concrete Beams Repaired/Reinforced by TRC Composites. Compos. Struct..

[19] Verbruggen S., Aggelis D.G., Tysmans T., Wastiels J. (2014). Bending of Beams Externally Reinforced with TRC and CFRP Monitored by DIC and AE. Compos. Struct..

[20] Elsanadedy H.M., Almusallam T.H., Alsayed S.H., Al-Salloum Y.A. (2013). Flexural Strengthening of RC Beams Using Textile Reinforced Mortar—Experimental and Numerical Study. Compos. Struct..

[21] Carozzi F.G., Milani G., Poggi C. (2014). Mechanical Properties and Numerical Modeling of Fabric Reinforced Cementitious Matrix (FRCM) Systems for Strengthening of Masonry Structures. Compos. Struct..

[22] D’Ambrisi A., Feo L., Focacci F. (2013). Experimental Analysis on Bond between PBO-FRCM Strengthening Materials and Concrete. Compos. Part B Eng..

[23] Kong K., Mesticou Z., Michel M., Si Larbi A., Junes A. (2017). Comparative Characterization of the Durability Behaviour of Textile-Reinforced Concrete (TRC) under Tension and Bending. Compos. Struct..

[24] Hegger J., Voss S. (2008). Investigations on the Bearing Behaviour and Application Potential of Textile Reinforced Concrete. Eng. Struct..

[25] de Andrade Silva F., Zhu D., Mobasher B. (2011). Impact Behavior of Sisal Fiber Cement Composites under Flexural Load. ACI Mater. J..

[26] Pakravan H.R., Jamshidi M., Rezaei H. (2016). Effect of Textile Surface Treatment on the Flexural Properties of Textile-Reinforced Cementitious Composites. J. Ind. Text..

[27] Tsesarsky M., Peled A., Katz A., Anteby I. (2013). Strengthening Concrete Elements by Confinement within Textile Reinforced Concrete (TRC) Shells—Static and Impact Properties. Constr. Build. Mater..

[28] Wu G., Wang X., Wu Z., Dong Z., Zhang G. (2015). Durability of Basalt Fibers and Composites in Corrosive Environments. J. Compos. Mater..

[29] Larrinaga P., Chastre C., Biscaia H.C., San-José J.T. (2014). Experimental and Numerical Modeling of Basalt Textile Reinforced Mortar Behavior under Uniaxial Tensile Stress. Mater. Des..

[30] Rambo D.A.S., de Andrade Silva F., Toledo Filho R.D., da Fonseca Martins Gomes O. (2015). Effect of Elevated Temperatures on the Mechanical Behavior of Basalt Textile Reinforced Refractory Concrete. Mater. Des. 1980–2015.

[31] Zhu D., Gencoglu M., Mobasher B. (2009). Low Velocity Flexural Impact Behavior of AR Glass Fabric Reinforced Cement Composites. Cem. Concr. Compos..

[32] Morelli F., Amico C., Salvatore W., Squeglia N., Stacul S. (2017). Influence of Tension Stiffening on the Flexural Stiffness of Reinforced Concrete Circular Sections. Materials.

[33] Meskenas A., Kaklauskas G., Daniunas A., Bacinskas D., Jakubovskis R., Gribniak S., Gelazius V. (2014). Determination of the Stress–Crack Opening Relationship of SFRC by an Inverse Analysis. Mech. Compos. Mater..

[34] Gribniak V., Ng P.-L., Tamulenas V., Misiūnaitė I., Norkus A., Šapalas A. (2019). Strengthening of Fibre Reinforced Concrete Elements: Synergy of the Fibres and External Sheet. Sustainability.

[35] Donnini J., Corinaldesi V., Nanni A. (2016). Mechanical Properties of FRCM Using Carbon Fabrics with Different Coating Treatments. Compos. Part B Eng..

[36] Hartig J., Häußler-Combe U. A model for the uniaxial tensile behaviour of Textile Reinforced Concrete (TRC) covering effects at the micro and meso scales. Proceedings of the ECF18.

[37] Häußler-Combe U., Hartig J. (2007). Bond and Failure Mechanisms of Textile Reinforced Concrete (TRC) under Uniaxial Tensile Loading. Cem. Concr. Compos..

[38] Häußler-Combe U., Jesse F., Curbach M. Textile Reinforced Concrete-Overview, Experimental and Theoretical Investigations. Fracture Mechanics of Concrete Structures, Proceedings of the Fifth International Conference on Fracture Mechanics of Concrete and Concrete Structures, Ia-FraMCos, Vail, CO, USA, 12–16 April 2004.

[39] Fowler D.W. (1999). Polymers in Concrete: A Vision for the 21st Century. Cem. Concr. Compos..

[40] ACI Committee 548 (2009). Guide for the Use of Polymers in Concrete, ACI 548. IR-09.

[41] Chmielewska B., Czarnecki L., Sustersic J., Zajc A. (2006). The Influence of Silane Coupling Agents on the Polymer Mortar. Cem. Concr. Compos..

[42] Chrissafis K., Bikiaris D. (2011). Can Nanoparticles Really Enhance Thermal Stability of Polymers? Part I: An Overview on Thermal Decomposition of Addition Polymers. Thermochim. Acta.

[43] Liang J.-Z. (2013). Reinforcement and Quantitative Description of Inorganic Particulate-Filled Polymer Composites. Compos. Part B Eng..

[44] Aghazadeh Mohandesi J., Refahi A., Sadeghi Meresht E., Berenji S. (2011). Effect of Temperature and Particle Weight Fraction on Mechanical and Micromechanical Properties of Sand-Polyethylene Terephthalate Composites: A Laboratory and Discrete Element Method Study. Compos. Part B Eng..

[45] Boudenne A., Ibos L., Fois M., Majesté J.C., Géhin E. (2005). Electrical and Thermal Behavior of Polypropylene Filled with Copper Particles. Compos. Part Appl. Sci. Manuf..

[46] Takahashi S., Imai Y., Kan A., Hotta Y., Ogawa H. (2014). Dielectric and Thermal Properties of Isotactic Polypropylene/Hexagonal Boron Nitride Composites for High-Frequency Applications. J. Alloys Compd..

[47] Danes F., Garnier B., Dupuis T. (2003). Predicting, Measuring, and Tailoring the Transverse Thermal Conductivity of Composites from Polymer Matrix and Metal Filler. Int. J. Thermophys..

[48] Wereszczak A.A., Morrissey T.G., Volante C.N., Farris P.J., Groele R.J., Wiles R.H., Wang H. (2013). Thermally Conductive MgO-Filled Epoxy Molding Compounds. IEEE Trans. Compon. Packag. Manuf. Technol..

[49] Olalla B., Carrot C., Fulchiron R., Boudimbou I., Peuvrel-disdier E. (2012). Analysis of the Influence of Polymer Viscosity on the Dispersion of Magnesium Hydroxide in a Polyolefin Matrix. Rheol. Acta.

[50] Cavodeau F., Otazaghine B., Sonnier R., Lopez-Cuesta J.-M., Delaite C. (2016). Fire Retardancy of Ethylene-Vinyl Acetate Composites—Evaluation of Synergistic Effects between ATH and Diatomite Fillers. Polym. Degrad. Stab..

[51] Mechanical Properties of Mg(OH)2/Polypropylene Composites Modified by Functionalized Polypropylene-Mai-2003-Journal of Applied Polymer Science-Wiley Online Library. https://onlinelibrary.wiley.com/doi/full/10.1002/app.11762.

[52] Tanaka T., Kozako M., Okamoto K. (2012). Toward High Thermal Conductivity Nano Micro Epoxy Composites with Sufficient Endurance Voltage. J. Int. Counc. Electr. Eng..

[53] Xia T., Cao Y., Oyler N.A., Murowchick J., Liu L., Chen X. (2015). Strong Microwave Absorption of Hydrogenated Wide Bandgap Semiconductor Nanoparticles. ACS Appl. Mater. Interfaces.

[54] Reis J.M.L., Ferreira A.J.M. (2003). The Influence of Notch Depth on the Fracture Mechanics Properties of Polymer Concrete. Int. J. Fract..

[55] Reis J.M.L., Ferreira A.J.M. (2004). Assessment of Fracture Properties of Epoxy Polymer Concrete Reinforced with Short Carbon and Glass Fibers. Constr. Build. Mater..

[56] Yu P., Manalo A., Ferdous W., Abousnina R., Salih C., Heyer T., Schubel P. (2021). Investigation on the Physical, Mechanical and Microstructural Properties of Epoxy Polymer Matrix with Crumb Rubber and Short Fibres for Composite Railway Sleepers. Constr. Build. Mater..

[57] Guerini V., Conforti A., Plizzari G., Kawashima S. (2018). Influence of Steel and Macro-Synthetic Fibers on Concrete Properties. Fibers.

[58] Khotbehsara M.M., Manalo A., Aravinthan T., Ferdous W., Benmokrane B., Nguyen K.T.Q. (2021). Synergistic Effects of Hygrothermal Conditions and Solar Ultraviolet Radiation on the Properties of Structural Particulate-Filled Epoxy Polymer Coatings. Constr. Build. Mater..

[59] Murcia D.H., Abdellatef M., Genedy M., Taha M.M.R. (2021). Rheological Characterization of Three-Dimensional-Printed Polymer Concrete. Mater. J..

[60] ASTM C1437-20, C01 Committee (2020). Test Method for Flow of Hydraulic Cement Mortar.

[61] Rebeiz K.S. (1995). Time-Temperature Properties of Polymer Concrete Using Recycled PET. Cem. Concr. Compos..

[62] Rebeiz K.S. (1996). Precast Use of Polymer Concrete Using Unsaturated Polyester Resin Based on Recycled PET Waste. Constr. Build. Mater..

[63] Tawfik M.E., Eskander S.B. (2006). Polymer Concrete from Marble Wastes and Recycled Poly(Ethylene Terephthalate). J. Elastomers Plast..

[64] Ohama Y., Demura K. (1982). Relation between Curing Conditions and Compressive Strength of Polyester Resin Concrete. Int. J. Cem. Compos. Lightweight Concr..

[65] ASTM C39-17b, C09 Committee (2017). Standard Test Method for Compressive Strength of Cylindrical Concrete Specimens.

[66] Du Y., Zhang X., Liu L., Zhou F., Zhu D., Pan W. (2018). Flexural Behaviour of Carbon Textile-Reinforced Concrete with Prestress and Steel Fibres. Polymers.

[67] Halvaei M., Jamshidi M., Latifi M., Ejtemaei M. (2020). Experimental Investigation and Modelling of Flexural Properties of Carbon Textile Reinforced Concrete. Constr. Build. Mater..

[68] Williams Portal N., Nyholm Thrane L., Lundgren K. (2016). Flexural Behaviour of Textile Reinforced Concrete Composites: Experimental and Numerical Evaluation. Mater. Struct..

[69] Başyiğit C., Çomak B., Kılınçarslan Ş., Serkan Üncü İ. (2012). Assessment of Concrete Compressive Strength by Image Processing Technique. Constr. Build. Mater..

[70] Mantawy I., Chennareddy R., Genedy M., Taha M.R. (2019). Polymer Concrete for Bridge Deck Closure Joints in Accelerated Bridge Construction. Infrastructures.

[71] Vipulanandan C., Dharmarajan N., Ching E. (1988). Mechanical Behaviour of Polymer Concrete Systems. Mater. Struct..

[72] Toufigh V., Hosseinali M., Shirkhorshidi S.M. (2016). Experimental Study and Constitutive Modeling of Polymer Concrete’s Behavior in Compression. Constr. Build. Mater..

[73] Teramoto A., Watanabe M., Murakami R., Ohkubo T. (2018). Visualization of Internal Crack Growth Due to Alkali–Silica Reaction Using Digital Image Correlation. Constr. Build. Mater..

[74] Carloni C., Subramaniam K.V. (2013). Investigation of Sub-Critical Fatigue Crack Growth in FRP/Concrete Cohesive Interface Using Digital Image Analysis. Compos. Part B Eng..

[75] Alam S.Y., Loukili A., Grondin F. (2012). Monitoring Size Effect on Crack Opening in Concrete by Digital Image Correlation. Eur. J. Environ. Civ. Eng..

[76] Kytinou V.K., Chalioris C.E., Karayannis C.G. (2020). Analysis of Residual Flexural Stiffness of Steel Fiber-Reinforced Concrete Beams with Steel Reinforcement. Materials.

[77] Choi S.-W., Choi J., Lee S.-C. (2019). Probabilistic Analysis for Strain-Hardening Behavior of High-Performance Fiber-Reinforced Concrete. Materials.

[78] Heras Murcia D., Minnig C., Gisiger J., Rösli U., Stormont J.C., Reda Taha M. Nano Sealant for CO_2_ Seal Integrity and Overcoring at Mont Terri. Proceedings of the 15th International Conference on Greenhouse Gas Control Technologies, GHGT-15.

